# Improving ECG Services at a Children's Hospital: Implementation of a Digital ECG System

**DOI:** 10.1155/2015/697149

**Published:** 2015-09-14

**Authors:** Frank A. Osei, Gregory J. Gates, Steven J. Choi, Daphne T. Hsu, Robert H. Pass, Scott R. Ceresnak

**Affiliations:** ^1^Division of Pediatric Cardiology, The Children's Hospital at Montefiore, Albert Einstein College of Medicine, 3415 Bainbridge Avenue, Bronx, NY 10467, USA; ^2^Division of Pediatric Cardiology, Stanford University, 725 Welch Road, MC 5912, Palo Alto, CA 94304, USA

## Abstract

*Background.* The use of digital ECG software and services is becoming common. We hypothesized that the introduction of a completely digital ECG system would increase the volume of ECGs interpreted at our children's hospital. *Methods.* As part of a hospital wide quality improvement initiative, a digital ECG service (MUSE, GE) was implemented at the Children's Hospital at Montefiore in June 2012. The total volume of ECGs performed in the first 6 months of the digital ECG era was compared to 18 months of the predigital era. Predigital and postdigital data were compared via *t*-tests. *Results.* The mean ECGs interpreted per month were 53 ± 16 in the predigital era and 216 ± 37 in the postdigital era (*p* < 0.001), a fourfold increase in ECG volume after introduction of the digital system. There was no significant change in inpatient or outpatient service volume during that time. The mean billing time decreased from 21 ± 27 days in the postdigital era to 12 ± 5 days in the postdigital era (*p* < 0.001). *Conclusion. *Implementation of a digital ECG system increased the volume of ECGs officially interpreted and reported.

## 1. Introduction

The clinical utility of the electrocardiogram (ECG) has changed markedly since the initial recordings of an ECG were made by Einthoven in the late 1890s [1]. The ECG has now become a standard and commonly used diagnostic tool that is employed by practitioners in all fields of pediatrics, from the private practice setting to major academic children's hospitals. ECGs are now commonly performed in the inpatient and outpatient settings to evaluate for congenital heart disease, to assess for arrhythmias, to evaluate cardiac symptoms, and to screen for cardiovascular diseases [2–9].

The classic pediatric electrocardiogram involves 15 leads (more than Einthoven's 3-lead system or the standard adult 12-lead ECG) and traditionally has been recorded by a paper copy that is usually interpreted by the ordering physician and then officially interpreted by a pediatric cardiologist. Because ECGs and ECG interpretation have historically been paper based, there is usually a delay between the time when the technician obtains the ECG and when the ECG is officially interpreted by a cardiologist for the formal medical record. In addition, depending on the practice setting, providers occasionally need to fax a copy of the electrocardiogram to the interpreting cardiologist and the fax process can significantly affect the quality of ECG and has the potential to affect the accuracy of the interpretation [10]. Today, many of these limitations still exist throughout institutional and private practice settings.

With rapidly evolving digital technology, electronic ECG systems have started to become widely used in major academic centers. The implementation of such systems is believed to improve the quality, accuracy, efficiency, and accessibility of ECG studies. There is little data in the published literature, however, assessing the implementation of such a system at a major children's hospital. The purpose of this study, therefore, was to review the implementation of a digital ECG system in the Division of Pediatric Cardiology of the Children's Hospital at Montefiore and compare the digital system to the previous traditional “paper” system. We hypothesized that implementation of a digital ECG system would increase the total number of ECGs officially interpreted and stored in the medical record. As a secondary outcome measure, we sought to determine if the digital ECG system would decrease the amount of time between ECG acquisition and billing for these professional services.

## 2. Methods

Approval for this study was obtained by the Institutional Review Board at Montefiore Medical Center. As a hospital wide quality improvement initiative, a new digital ECG system (MUSE©, GE) was implemented at the Children's Hospital at Montefiore (CHAM), Albert Einstein College of Medicine, in June 2012. This system provides high-fidelity digital access to electrocardiography data, allowing the clinician to review and edit studies online instantaneously after an ECG is performed and this process is described in greater detail below. For the purpose of this case-control investigation, the postdigital ECG era (June 2012 through December 2012, 6-month period) was compared to the predigital ECG control era (January 2011 through June 2013, 18-month period). The primary outcome measure was the total number of ECGs officially interpreted, documented in the medical record, and billed per month. The secondary outcome measures included the time intervals between ECG acquisition, interpretation, and billing for professional services.

### 2.1. Predigital ECG Era Workflow

**Figure 1:**
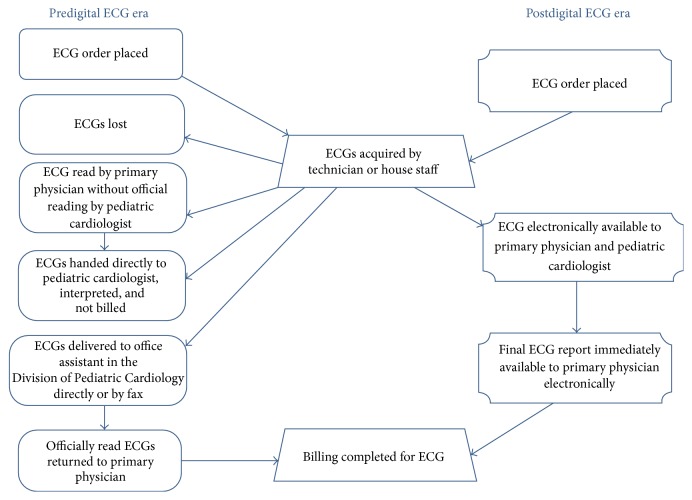
ECG workflow in the predigital versus digital eras.

**Figure 2:**
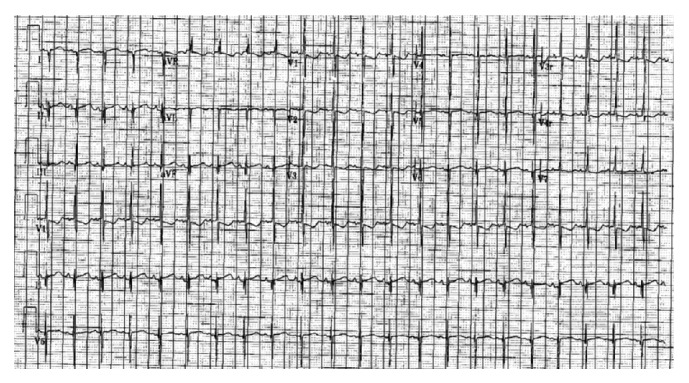
A copy of a faxed ECG.

In the busy tertiary hospital inpatient setting, many ECGs are ordered, performed, and interpreted at the bedside or in the office without adherence to strict guidelines. This may be due to human factors, acuity of patients, or immediate availability of the cardiologist to provide immediate interpretation of the ECGs. In the predigital era, ECGs that were performed in the Children's Hospital at Montefiore (CHAM) were sent to an office assistant in the Division of Pediatric Cardiology, who would create a billing sheet, attach the billing sheet to the ECG, and then send the ECG to be officially read by the assigned interpreting cardiologist. After the cardiologist had interpreted the ECG, it was sent back to the office assistant who then sent it back to the provider ordering the ECG through interoffice mail (Figure 1). The billing sheet was then sent to another office for official billing. The ECG was then sent to medical records where it was scanned into the electronic medical record for future reference and review. In addition, many ECGs were often performed by other healthcare providers (such as residents and fellows on the inpatient wards) without formal interpretation and documentation of the study by the pediatric cardiology team. ECGs that were also performed in affiliated hospitals and the neonatal intensive care units (located at multiple medical campuses) were initially faxed for urgent interpretation and then also sent through interoffice mail for official interpretation for the medical record. Figure 1 illustrates the complex and often variable processes involved in the predigital ECG era. The complex process of acquisition through ECG interpretation of a paper ECG was fraught with the opportunity for a multiple problems, including a delay in official interpretation or loss of ECGs in transit. As previously discussed, ECGs were often faxed to the cardiologist and the ability and accuracy of the interpretation were limited [10]. An example of such faxed ECGs is illustrated in Figure 2. In addition to fax distortion of the ECG and difficulties with interpretation, there were also technical limitations with this process including technical fax machine snags, faxes being sent incorrectly, and the need to physically go to a fax machine to access the faxed ECG. The official report would then be faxed back with a written report, adding an additional potential challenge for the ordering physician. Sometimes the handwriting of the interpreting cardiologist may not be easily legible which can affect the quality of patient care. In an effort to improve patient care and increase efficiency in the ECG delivery system, the digital ECG system was introduced at the Children's Hospital at Montefiore and its related pediatric care hospitals and neonatal intensive care units (Montefiore North Division and Weiler Hospital).

### 2.2. Postdigital ECG System Workflow

**Figure 3:**
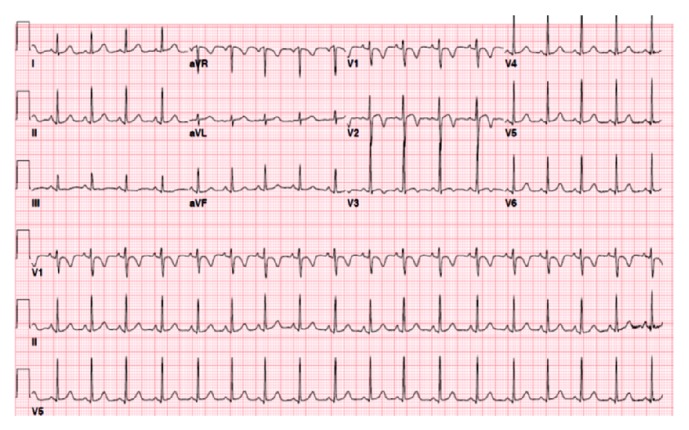
A copy of ECG acquired during the digital era.

After adoption of a digital ECG system, the workflow process for pediatric ECGs changed markedly as detailed in Figure 1. All ECG carts were built with wireless capabilities and all ECGs performed were automatically uploaded to the MUSE© (GE) system for review. Before an ECG was performed, an order was placed for ECG electronically in the hospital electronic medical record system. The barcode on the patient's wrist identification band was scanned by a barcode scanner on the ECG cart which linked the order for the ECG with the MUSE system. If an emergent ECG was needed or barcode scanning could not be done, manual inputs for patient identifiers could always be performed and the patient information could be edited at a later time. Any ECG that was performed was then stored on the digital server and became immediately available online for review by a pediatric cardiologist. All ECGs performed at CHAM and some of the CHAM affiliated sites (Weiler Hospital and Montefiore North Division) became electronically stored and immediately available for review by any practitioner with access to the MUSE system. The ECGs could be viewed on a viewing platform that was password protected and encrypted and so patients' information was protected. The ECGs were available for review and interpretation both in the hospital and through a secure HIPPA compliant remote server. As a result, ECGs could be immediately read with an official report immediately transmitted onto the electronic medical record. The quality of such digital ECGs is illustrated in Figure 3.

### 2.3. Data and Statistical Analysis

Statistical analysis was performed using SPSS software (IBM, Armonk, NY). Categorical and dichotomous variables were expressed as percentages and continuous variables were expressed as mean ± standard deviation or median (range). The total numbers of ECGs performed per month in the predigital era (control group) were compared to the number of ECGs performed in the postdigital era via *t*-test. The time from ECG acquisition to billing for the ECG service was compared between the predigital and postdigital eras via *t*-test. Though billing ultimately became digitalized, during the first 6 months of the digital era, the prior predigital era billing practice was similar to the digital era. The time from ECG acquisition to interpretation was calculated for the digital era. Unfortunately, the exact date and time of ECG interpretation were not ascertainable in the predigital era so no comparison of interpretation time between eras could be performed. To help assess the impact and potential confounder of hospital growth and program expansion during this time period, total inpatient and outpatient volume in CHAM and in the Division of Pediatric Cardiology in the pre- and postdigital eras were assessed via control charts and compared between the pre- and postdigital eras via ANOVA. All *p* values < 0.05 were considered statistically significant.

## 3. Results

### 3.1. ECG Interpretation Volumes

**Figure 4:**
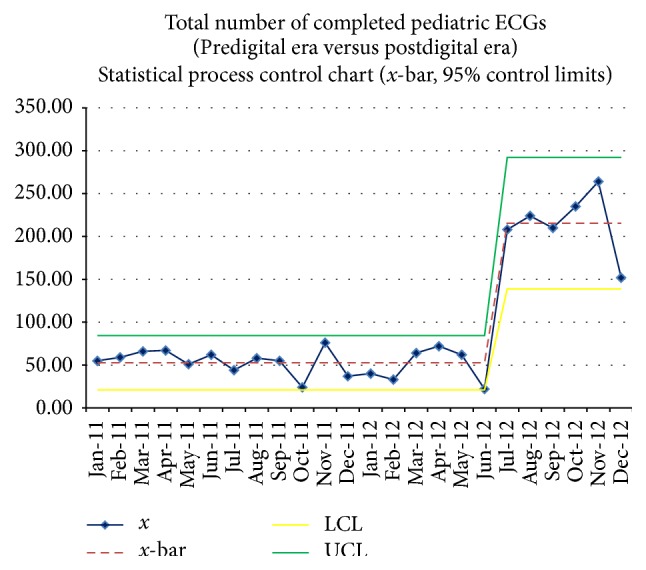
Total number of ECGs read over the study period. This quality control chart demonstrates the marked increase in the volume of ECGs that were interpreted with the implementation of digital ECG services in June 2012 (*x*—number of ECGs, *x*-bar—mean, LCL—lower control limit, and UCL—upper control limit).

**Figure 5:**
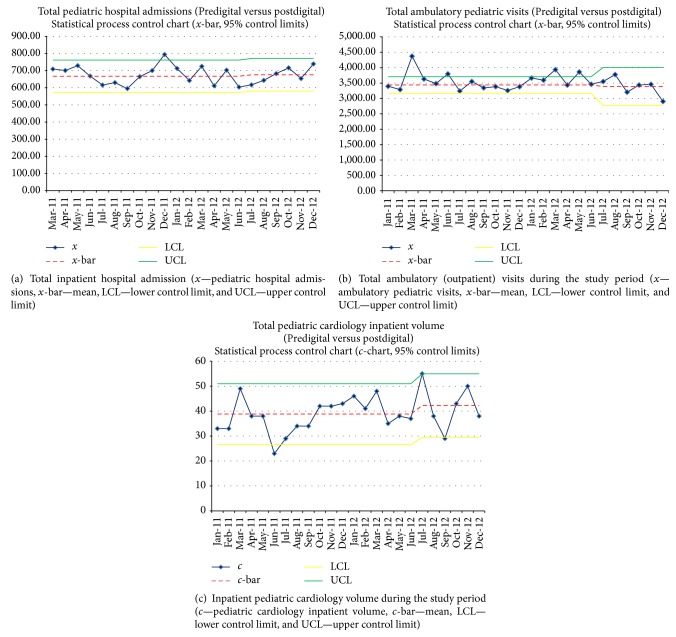
Hospital inpatient and outpatient volumes during the study period. These figures demonstrate that while the total number of ECGs interpreted after the introduction of the digital ECG system markedly increased, there was no significant increase in total hospital admissions (a), total ambulatory visits (b), or inpatient pediatric cardiology volume (c) that could have accounted for that increase.

In the predigital era, the mean number of ECGs per month which were officially interpreted was 53 ± 16, while in the digital era the mean number of ECGs which were officially read was 216 ± 37 (*p* < 0.001), a fourfold increase in ECG volume after introduction of the digital ECG system.  Figure 4 illustrates the trend of the number of ECGs officially read during the predigital and the postdigital eras. The control chart demonstrates that the increased number of official monthly ECGs was not a result of random variation as evidenced by the new monthly mean being well above the upper control limit (95% control limits) of the prior predigital monthly mean. The total number of ECGs officially interpreted in the 18-month predigital era was less than the number of ECGs read in the 6-month digital era, with 947 ECGs interpreted from January 2011 to June 2012 and 1293 ECGs interpreted from June 2012 to December 2012. As illustrated in Figure 5, there was not a significant difference in overall inpatient hospital volume, inpatient cardiology volume, and outpatient visits over this time period. The mean number of monthly admissions in CHAM during the predigital and the digital eras was 673 ± 53 and 675 ± 46 (*p* = 0.92), respectively, the mean number of outpatient visits in each era was 3,560 ± 287 and 3,388 ± 302 (*p* = 0.22), and the mean number of monthly pediatric cardiology inpatient volumes during each era was 37.9 ± 6.6 and 40.1 ± 9.3, respectively (*p* = 0.23) (Table 1).

### 3.2. ECG Interpretation and Billing Times

After introduction of the digital ECG system, the mean time from ECG acquisition to interpretation was 26 ± 27 hours, with a median interpretation time of 17 hours [range 0.03–131]. The billing time was significantly decreased after introduction of the digital ECG system, with a mean billing time in the predigital era of 21 ± 27 days compared to 12 ± 5 days in the postdigital era (*p* < 0.001) (Table 1).

## 4. Discussion

In this investigation, we have demonstrated that implementation of a digital ECG system through a quality improvement initiative at the Children's Hospital at Montefiore resulted in a marked increase in the total volume of ECGs that were officially reviewed, interpreted, and billed by the Division of Pediatric Cardiology. In addition, the overall efficiency and medical-legal documentation were significantly improved and the billing time was markedly reduced with the implementation of the digital system.

**Figure 6:**
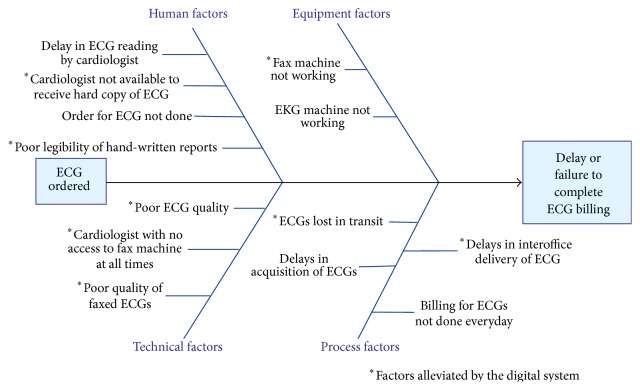
Fishbone diagram of factors affecting ECG workflow.

The etiology for the marked increase in the total volume of ECGs after implementation of the digital ECG system is likely multifactorial, though primarily related to use of digital ECG carts and a digital ECG server (MUSE system). The overall hospital and cardiology volumes did not significantly change during the implementation period, and the patient volume was therefore not the primary etiology of the marked increase in ECG volume. The increase in ECG volume was mainly due to the major intervention of implementing the digital ECG system with capture and routing of all ECGs into the digital ECG system. Also, there may not have been a significant change in the referral pattern or volume during the period; hence, the increase in volume was likely from capturing more ECGs in the system than increasing referral patterns. The change in workflow is demonstrated in Figure 1 and the challenges with respect to human, technical, processing, and equipment factors are displayed in the Fishbone diagram (Figure 6). As illustrated in Figure 1, some of the hard copies of ECGs performed in the predigital era may have been lost, while some of them may have been reviewed by the primary physician with or without input from the pediatric cardiologist. In the predigital era, there may have been many ECGs reviewed by the pediatric cardiologist but not formally interpreted for the medical record or not formally processed for billing. With the introduction of the digital system, the change in workflow enabled immediate ECG access, better medical and legal documentation of the ECG test, an immediate report in the medical record, and better catchment of all ECGs performed. This represents a fairly dramatic workflow improvement with fewer steps and a more streamlined and efficient process (Figure 1).

**Figure 7:**
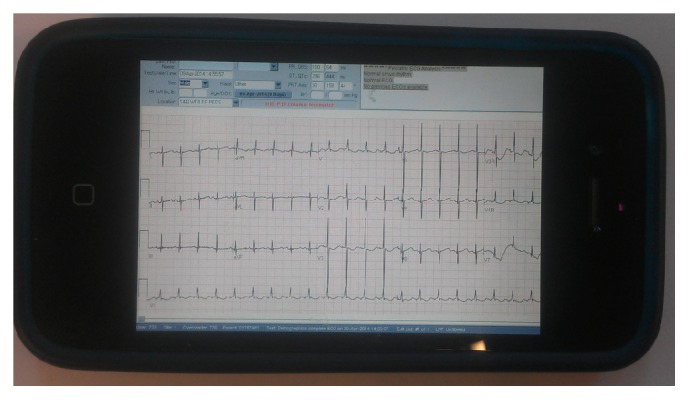
A photograph of a smartphone demonstrating a digital ECG.

In addition to improving the quantity of ECGs officially interpreted, the overall quality of the ECG for interpretation was improved. The digital quality of the ECGs electronically available for official reading was excellent and the system eliminated the inaccuracies associated with ECGs transmitted by fax [10]. ECGs stored within the digital system could be read by any of the authorized practitioners at any time, from any location within or outside the hospital or clinic setting. The ECGs could be viewed on a viewing platform that was password protected and encrypted and so patients' information was protected. ECGs could therefore be interpreted at any computer with safe and secure remote login and could also be accessed via a smartphone (Figure 7). Whenever an ECG was officially read by the cardiologist, an official report was immediately available for the primary physician to access via the digital system. In addition to the decrease in interpretation time, issues of poor legibility of the handwritten reports were also eliminated. The Fishbone diagram (Figure 6) illustrates the factors that were eliminated by the introduction of the digital system. These factors affected not only the number of the ECGs officially read but also the delay involved in the process from the acquisition of the ECG to the billing stage. In the predigital era, hard copies of ECGs delivered to the division of cardiology could not be read if the cardiologist was not readily available, but with the digital system, the cardiologist was able to access any ECG electronically from any location from within or outside of the hospital. Physicians could therefore provide faster diagnosis and treatment for their patients.

With the elimination of some of the processes that cause delays in official and accurate ECGs interpretation and timely reporting of result, the digital system increased the quality and efficiency of patient care and improved the electrocardiographic services provided by the division of pediatric cardiology. Even though the implementation of the digital system involves some financial investment, the numbers indicate that there is a theoretical increase in the revenue with better billing capture in the long run. The digital system has thus shown the potential of increasing the revenue generated by the division of pediatric cardiology through the official interpretation and billing of ECGs. In addition, digital systems offer the potential to ensure data accuracy, data accessibility, and data consistency. These systems can help track every ECG performed across the medical system and ensure that there is an order and official interpretation for each ECG performed. The digital ECG system therefore increased the productivity of the ECG services at the hospital, improved compliance with timely and official test interpretation and documentation, streamlined the data access and output processes of ECG interpretation, and improved the overall quality of ECG services in our children's hospital.

## 5. Limitations

Due to the retrospective nature of the investigation, the time interval between ECG acquisition and interpretation in the predigital era was not available and therefore no comparison could be made between ECG acquisition and interpretation time in the pre- and postdigital eras. The billing process during the first 6 months of the digital program did not markedly differ from the predigital era, though, and billing time was therefore used as a surrogate comparison. In addition, during the initial roll-out of the digital ECG system, it was noted that ECGs were occasionally getting “stuck” in the ECG machines and not getting transferred to the server to allow for review and interpretation. This was resolved by installing additional wireless features to the ECG carts. This delay in transmission may have led to overestimation of the time between ECG acquisition and ECG interpretation in the digital era. The time of follow-up was shorter in the postdigital era compared with the predigital era. Finally, the accuracy and completeness of the reports of the ECGs officially read during the two eras were not evaluated in this study.

## 6. Conclusion

The implementation of a digital ECG system in our Children's Hospital increased the total number of ECGs officially interpreted and reported. The ECGs were interpreted and reported in the EMR faster and the digital ECG system increased efficiency of ECG services.

## Figures and Tables

**Table 1:** Summary of results.

	Predigital mean ± standard deviation	Postdigital mean ± standard deviation	*p* value
ECGs interpreted per month	53 ± 16	216 ± 37	**<0.001**
Billing time (days)	21 ± 27	12 ± 5	**<0.001**
Monthly admissions in CHAM	673 ± 53	675 ± 46	0.92
Monthly ambulatory pediatric visits	3,560 ± 287	3,388 ± 302	0.22
Monthly pediatric cardiology inpatient volumes	37.9 ± 6.6	40.1 ± 9.3	0.23
